# A case of metachronous triple primary carcinoma complicated with pulmonary tuberculosis: Case report and review

**DOI:** 10.1097/MD.0000000000039638

**Published:** 2024-09-20

**Authors:** Ying Chen, Shu Luo, Quan Zheng, Qing Yu, Chunxia Liu, Rui Tang, Fei Chen, Yan Zhang

**Affiliations:** a Department of Oncology, Suining First People’s Hospital, Suining, China; b Department of Respiratory Medicine, Suining First People’s Hospital, Suining, China; c Department of Pathology, Suining First People’s Hospital, Suining, China; d Department of Lung Cancer Center, West China Hospital, Sichuan University, Chengdu, China.

**Keywords:** metachronous triple primary carcinoma, multiple primary neoplasms, P53, squamous cell carcinoma, tuberculosis

## Abstract

**Rationale:**

Multiple primary malignant neoplasms with tuberculosis are rare. The interaction between tuberculosis and tumor remains unclear. Moreover, the treatment of multiple primary tumors combined with tuberculosis is relatively complicated. Herein, we report a case of metachronous triple primary carcinoma complicated with pulmonary tuberculosis.

**Objective:**

This report aims to analyze the clinical characteristics of 3 primary tumors combined with tuberculosis. We report the long-term survival of this patient after personalized treatment and this patient have a good quality of life.

**Diagnoses and interventions:**

A 55-year-old male patient was diagnosed with squamous cell carcinoma of the lower thoracic esophagus (cT4bN1M0 IVA) and received concurrent chemoradiotherapy, followed by 2 cycles consolidate chemotherapy. During the follow-up, he was diagnosed with secondary tuberculosis (TB) and accepted anti-TB treatment. During anti-TB treatment, he was diagnosed with squamous cell carcinoma of the oropharynx (cT1N0M0 I P16(‐)), then he received radical radiation therapy. However, within a year, the patient was diagnosed with oral squamous cell carcinoma (cT3N0M0 IIIA). He accepted an individualized chemotherapy with paclitaxel combined with capecitabine. Moreover, immunohistochemistry of the patient’s 3 biopsies indicated positive P53 expression.

**Outcomes:**

Since the patient suffered from esophageal cancer, oropharyngeal cancer, and oral floor cancer, no tumor recurrence or metastasis was observed. And he has a good quality of life. Tuberculosis, TP53 mutation, radiotherapy, smoking, and drinking history may be risk factors for multiple primary tumors.

**Lessons:**

The treatment of multiple primary tumors combined with pulmonary tuberculosis is complicated. Individualized treatment allows patients to achieve long-term survival while also having a good quality of life. Limitations in this case: surgery may be an alternative strategy for the patient, but the patient refused surgery.

## 1. Introduction

With the progress of medical diagnosis and treatment, the survival of cancer patients is prolonged, and the incidence of multiple primary malignant neoplasms (MPMNs) is increasing.^[1]^ In epidemiological studies, the incidence of multiple primaries is reported to be in the range of 2% to 17%.^[2]^ In a Chinese retrospective study, MPMNs accounted for 1.12% of all malignancies (270/24,105).^[3]^ Triple cancer is reported to be in the range of only 0.03% to 0.16%.^[4,5]^ Moreover, MPMNs combined with pulmonary tuberculosis is rare. Therefore, we report a case of metachronous triple primary carcinoma complicated with pulmonary tuberculosis. From 2017 to 2022, the patient developed esophageal squamous cell carcinoma, tuberculosis, oropharyngeal cancer, and oral floor cancer. The patient is still alive after radiotherapy, chemotherapy, and antituberculosis therapy. At present, the pathogenesis and risk factors of multiple primary malignant tumors are still unclear. We report the long-term survival of this patient after treatment and review the relevant literature.

## 2. Case presentation

**Figure 1.**
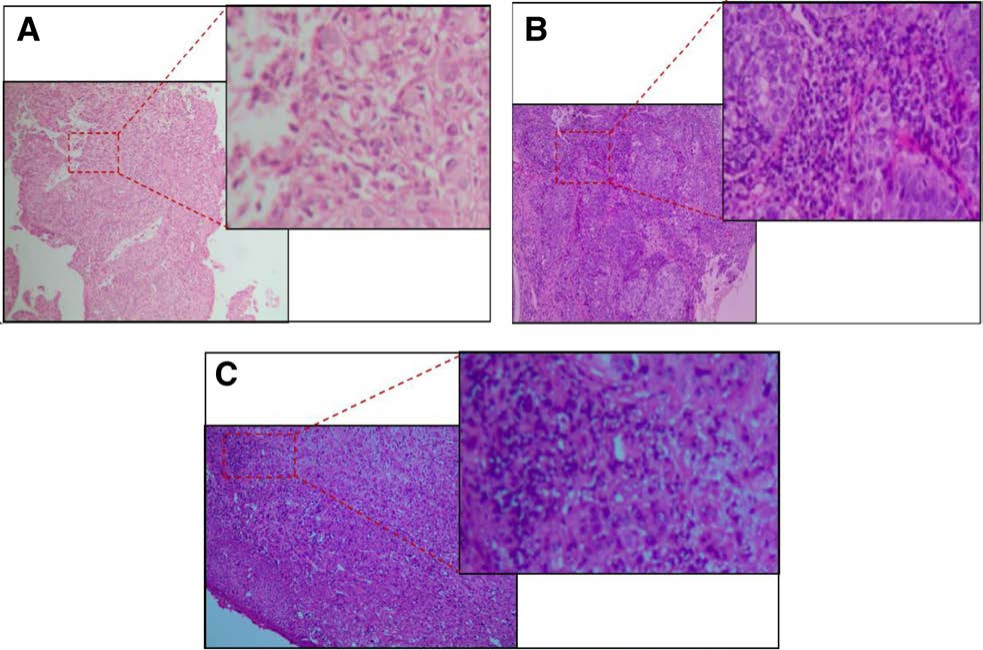
(A) Esophageal squamous cell carcinoma HE staining. (B) Oropharyngeal carcinoma HE staining. (C) Oral cancer HE staining.

**Figure 2.**
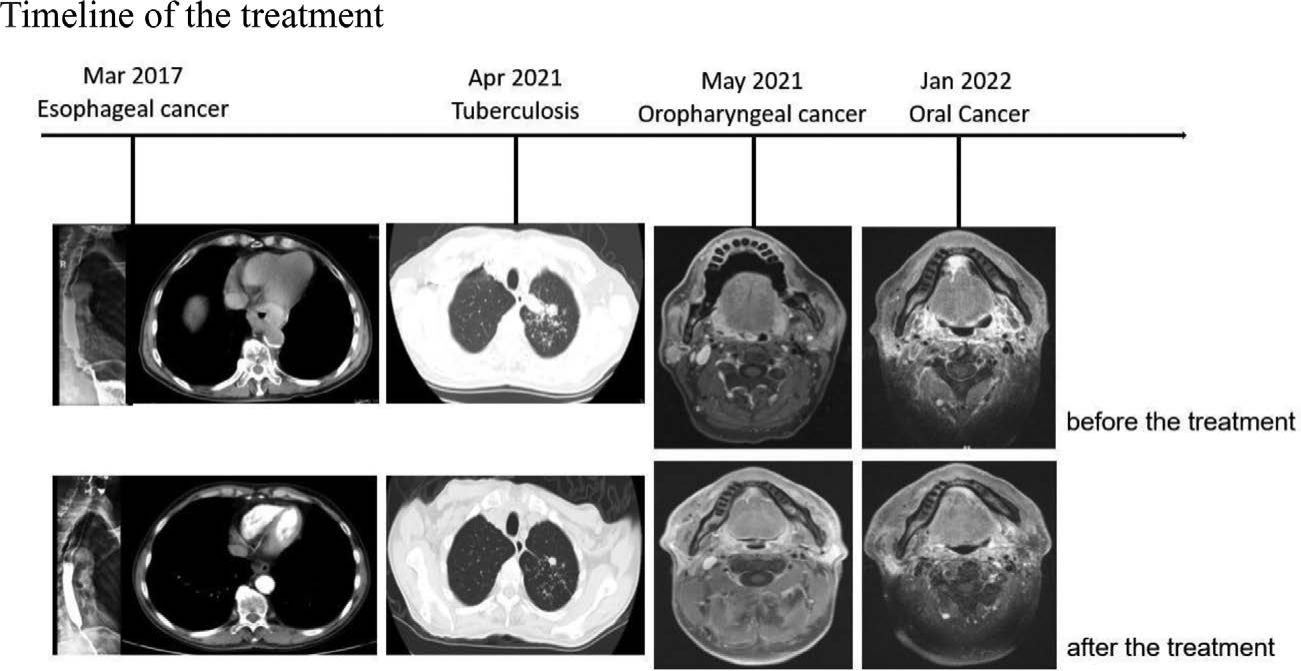
Comparison of esophageal cancer, pulmonary tuberculosis, oropharyngeal cancer, and oral cancer before and after treatment.

A 55-year-old man from Sichuan Province, China, was admitted to our hospital. In March 2017, with the complaint of swallowing obstruction for 3 months. He had a history of smoking and drinking for more than 30 years. Gastroscope was performed and showed neoplasm 35 cm from incisor, and a biopsy of the esophageal neoplasm confirmed squamous cell carcinoma (Fig. 1A). Upper digestive tract angiography showed T8–9 to the cardia, total length of about 8 cm esophagus wall uneven thickening. CT was performed to exclude metastasis and showed swollen lymph nodes near the cardia which the largest one has a short diameter of 1.4 cm. Accordingly, the patient was diagnosed with squamous cell carcinoma of the lower thoracic esophagus (cT4bN1M0, stage IVA by the American Joint Committee on Cancer [AJCC], 8th edition). The patient received concurrent chemoradiotherapy: 64Gy/32F/6 + w, DP (docetaxel 75 mg/m^2^ and cisplatin 75 mg/m^2^ q3w). Two cycles of synchronous chemotherapy followed by 2 cycles of consolidation chemotherapy (Fig. 2). His obstructive symptoms were markedly relieved. During the follow-up period, no local recurrence or distant metastasis was found. In April 2021, CT was performed and showed a infectious lesion at the apex of the left lung. He went to a hospital for infectious diseases and underwent a needle biopsy of the lung lesion. The results showed chronic granulomatous inflammation and acid-fast staining was found to be positive for mycobacterium. The result of T-SPOT was positive. Then he was diagnosed with secondary tuberculosis and accepted anti-tuberculosis (TB) treatment with 2-month HRZE (isoniazid–rifampin–pyrazinamide–ethambutol) followed by 8-month isoniazid-rifampin (HR) (Fig. 2). In May 2021, he has a sore throat, squamous cell carcinoma of the right side of the oropharynx was found by biopsy (Fig. 1B), then he came to our hospital, MRI of oropharynx and CT of thorax and abdomen was performed to reveal the stage. Accordingly, the patient was diagnosed with squamous cell carcinoma of the oropharynx (cT1N0M0 stage I P16(‐) by the AJCC, 8th edition). He received radical radiation therapy, then conformal radiation therapy was initiated with a total dose of 72 Gy in 36 fractions and MRI showed that the lesions became smaller after treatment (Fig. 2). Meanwhile, he accepted anti-TB treatment. However, in January 2022 he was admitted to our hospital, with the complaint of oral pain. Examination revealed new organisms on the floor of the mouth. The biopsy indicated squamous cell carcinoma (Fig. 1C). MRI of oral cavity and CT of thorax and abdomen was performed to reveal the stage. Accordingly, the patient was diagnosed with oral squamous cell carcinoma (cT3N0M0 stage IIIA by the AJCC, 8th edition). In consideration of various reasons including he refused surgery and he underwent head and neck radiation therapy within a year. Moreover, his creatinine is slightly elevated after anti-TB treatment. He accepted an individualized chemotherapy with paclitaxel combined with capecitabine: paclitaxel 135 mg/m^2^, ivgtt, d1, every 3 weeks, and capecitabine, 1250 mg/m^2^, po, bid, d1–14, every 3 weeks. He was treated with 6 cycles of chemotherapy. MRI showed that the tumor was smaller and less strengthened (Fig. 2). Then he fellow-up regularly. So far, the patient is still alive, with no local recurrence or distant metastasis was found in the 3 parts, and he has a good quality of life.

## 3. Discussion

In this case, we report a case of metachronous triple primary cancer combined with tuberculosis for the first time. At present, there are more and more reports on multiple primary cancers, but the pathogenesis of multiple primary malignancies is still unclear, and which factors may cause the increased risk of multiple primary tumors are worthy of further investigation. In addition, the relationship between tuberculosis and non-lung primary tumors and whether tuberculosis is associated with multiple primary cancers need further exploration.

In our case, tuberculosis occurred after esophageal cancer, and oropharyngeal and oral cancer occurred after tuberculosis infection. An 18-year retrospective cohort study in Korea shows that in all cancer cases, the incidence rate ratios of bacteriologically confirmed tuberculosis was 14.30.^[6]^ And another study showed that compared with the general population, head and neck cancer is associated with 2.90-fold increased hazard of pulmonary tuberculosis.^[7]^ Moreover, it is reported that the global population attributable fraction of tuberculosis due to cancer was 1.85% (95% CI, 1.77–1.97%) in 2019.^[8]^ And a population-based cohort study revealed that the risk of TB is higher in the year before and after cancer diagnosis.^[9]^ From these data, we can see that malignancy increases the risk of tuberculosis. At the same time, tuberculosis can also increase the risk of malignancy. A meta-analysis showed that tuberculosis was associated with increased risk of cancer at 10 sites, with the highest risk for head and neck cancer in particular (RR 2.64 [95% CI, 2.00–3.48]). It is important to note that China and India accounted for 47% of all tuberculosis-related cancer cases.^[10]^ And another meta-analysis revealed that the standardized incidence ratios of all cancer was highest within the first year following TB (SIR 4.70, 95% CI, 1.80–12.27, *I*^2^ = 99%).^[11]^ Data from the Taiwan National Health Insurance database from 2000 to 2010 were analyzed and the probability of cancer in TB patients was 2.07 times higher than that in general patients (2.07, 95% CI, 1.90–2.26). For male, the risk of head and neck, esophageal, and colorectal cancers increases within 1 year of TB diagnosis.^[12]^ It is reported that risk of most non-pulmonary cancers was increased 3-fold within the first year following diagnosis of tuberculosis.^[13]^ From the above studies, we can find that tuberculosis can increase the risk of non-pulmonary primary malignant tumors, and the risk of tuberculosis is higher within 1 year after tuberculosis. In our case, cancer of the oropharynx and floor of the mouth was found in the patient within the first year after diagnosis of tuberculosis. For patients with tuberculosis, effective screening strategies may help in the early detection of malignancies. However, the related mechanism of malignant tumor after tuberculosis is still unclear, so the mechanism of malignant tumor after tuberculosis needs to be further explored.

In this case, the patient was a heavy smoker and drinker. In the cause of cancer, tobacco is the main carcinogen, ethanol association Simultaneous action promotes carcinogenesis. It was reported that 86.4% of patients with esophageal cancer-related head and neck cancer have a history of smoking and alcohol consumption.^[14]^ And Anouk Overwater’s study showed that in Western patients with head and neck squamous cell carcinoma, alcohol was significant predictors for esophageal second primary tumors (SPCs).^[15]^ However, compared with never smokers, ever smokers had 59% and 102% higher risks for all SPCs and smoking-related SPCs, respectively.^[16]^ These findings are useful in encouraging smokers to quit and in preventing never-smokers from starting to smoke, as well as for drinkers. In this case, the patient was a heavy smoker and alcohol drinker, and the correlation between smoking and alcohol consumption and the occurrence of multiple primary cancers in this patient is not clear. However, the theory of field cancerization further supports the importance of environmental factors in multiple primary cancers.^[17]^ Therefore, the history of smoking and drinking may be involved in the occurrence of multiple primary cancers in this case.

In our case, the patient received radiation therapy twice. The radiation (γ rays) of radiation therapy is a carcinogen, and the surrounding normal tissues will inevitably be irradiated and cause DNA damage, so the risk of secondary tumor development in the radiation area and its surrounding tissues is also increased. A pooled analysis of 9 cancer registries revealed that among all esophageal cancer survivors, radiotherapy increased the risk of second primary cancers including laryngeal (SIR 3.98, 95% CI, 2.43–6.14) and thyroid (SIR 3.57, 95% CI = 1.54–7.03).^[18]^ Surprisingly, a population-based analysis in Taiwan pointed out that for breast cancer patients, the risk of developing second primary lung cancer was 10.078 times higher in the radiotherapy group than in the non-radiotherapy group.^[19]^ However, the risk of developing a second primary cancer is related to the radiation dose, and radiation-associated second primary tumors usually occur decades after radiation therapy.^[20,21]^ The patient received radiation therapy twice, but the time did not reach the incubation period reported in the literature. Long-term survival of patients is achieved through individualized treatment. In the follow-up, attention should be paid to the radiotherapy area and surrounding area of the patient and we should be cautious of secondary malignancy occurs again.

**Figure 3.**
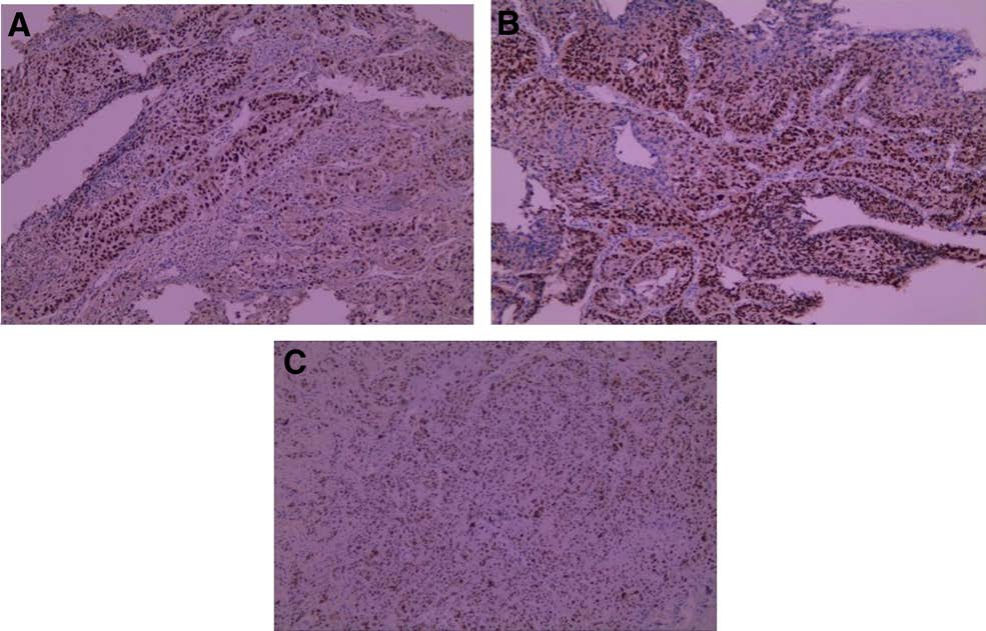
(A) Positive P53 in esophageal squamous cell carcinoma. (B) Positive P53 in oropharyngeal squamous cell carcinoma. (C) Positive P53 in oral floor squamous cell carcinoma.

It is also worth noting that immunohistochemical tests for 3 primary cancers in this case indicated positive P53 (Fig. 3). The P53 protein is related to the P53 gene. Genetic factors are also an important factor in the development of multiple primary cancers. Based on an analysis of 1191 cancer index patients attending the NCIS Cancer Genetics Clinic, the results of a multigenomic clinical genetic test in Asian patients with multiple primary cancers showed that, in patients with multiple primary cancers, pathogenic variants include BRCA1, BRCA2, mismatch repair genes, APC, ATM, MUTYH, PALB2, RAD50, and TP53 were found.^[22]^ However, in this study, multiple primary tumors mainly involved breast cancer, ovarian cancer, colon cancer, and so on. Whereas, in this case, most of the primary tumors were esophageal cancer and head and neck cancer. Studies have shown that in head and neck cancer, the P53 mutation rate exceeds 50%.^[23]^ The analysis of mutated genes in synchronous multiple primary carcinoma suggests that P53 and PKB signaling pathways may be involved in the occurrence of synchronous multiple primary carcinoma.^[24]^ Another study indicated that p53 Codon 72 Polymorphism were associated with the risk of second primary tumors in patients with head and neck squamous cell carcinoma. Compared with SCCHN patients with the p53 72Arg/Arg genotype, there was a significantly greater risk of SPM associated with the p53 72Arg/Pro genotype (hazard ratio [HR], 1.75; 95% CI, 1.17–2.61) and combined p53 72Arg/Pro þ Pro/Pro (HR, 1.58; 95% CI, 1.07–2.34).^[25]^ Although the relationship between P53 gene and multiple primary cancers is still unclear, the immunohistochemistry of the 3 biopsies of this patient in our case all showed positive P53, suggesting that it may also be related to multiple primary cancers. It is a pity, the patient did not undergo genetic testing for financial reasons.

## 4. Conclusion

Herein, we report a case of metachronous triple primary cancer with pulmonary tuberculosis. The immunohistochemical P53 of the 3 tumors in this case was positive, suggesting that TP53 mutation may be highly correlated with the occurrence of multiple primary tumors in the patient, and the history of smoking, drinking, and radiotherapy may be high-risk factors for its occurrence, and tuberculosis may be related to the occurrence of multiple primary cancers. Considering the effect of antituberculosis treatment on tumor treatment, the treatment of patients with multiple primary tumors complicated with tuberculosis is more complicated. The choice of individual treatment options enables patients to achieve a good quality of life while achieving long-term survival.

## Acknowledgments

The authors sincerely thank Mr. Yanyang Liu for language assistance.

## Author contributions

**Supervision:** Shu Luo, Fei Chen.

**Writing – original draft:** Ying Chen.

**Writing – review & editing:** Quan Zheng, Qing Yu, Chunxia Liu, Rui Tang, Yan Zhang.
